# Supplementary figures and images for: Position effects influencing intrachromosomal repair of a double-strand break in budding yeast
Source: PLoS One. 2017 Jul 11;12(7):e0180994. doi: 10.1371/journal.pone.0180994 (PMC5507452; doi:10.1371/journal.pone.0180994)

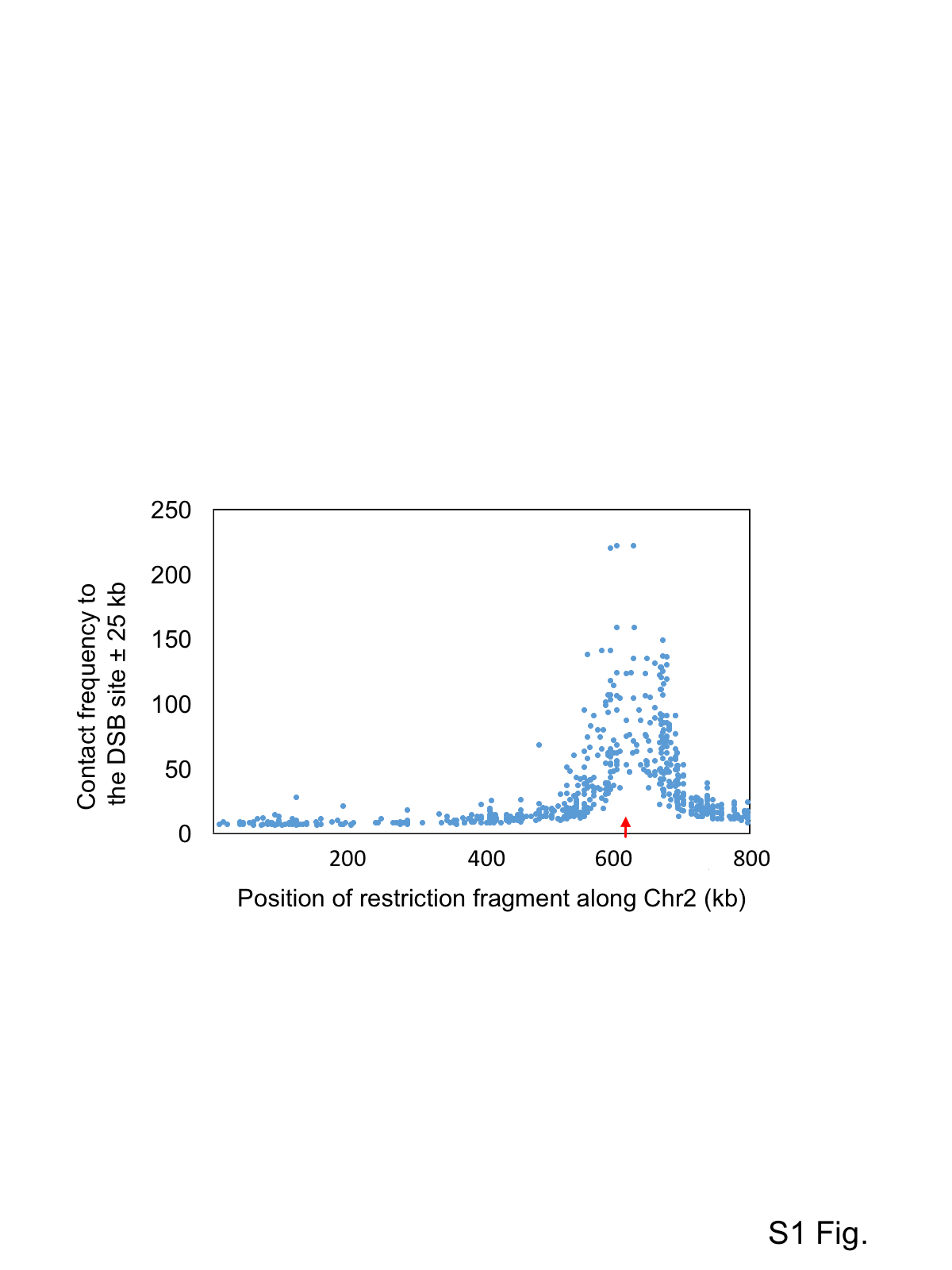

Supplement: S1 Fig — The contacts were calculated using ±25 kb window size around the DSB (Chr2, 625 kb). The contact frequency between the DSB and the donor is determined by adding up all individual contacts around the donor location. The position of the HO cleavage site is given by a red arrow. (TIF) [file pone.0180994.s001.tif]

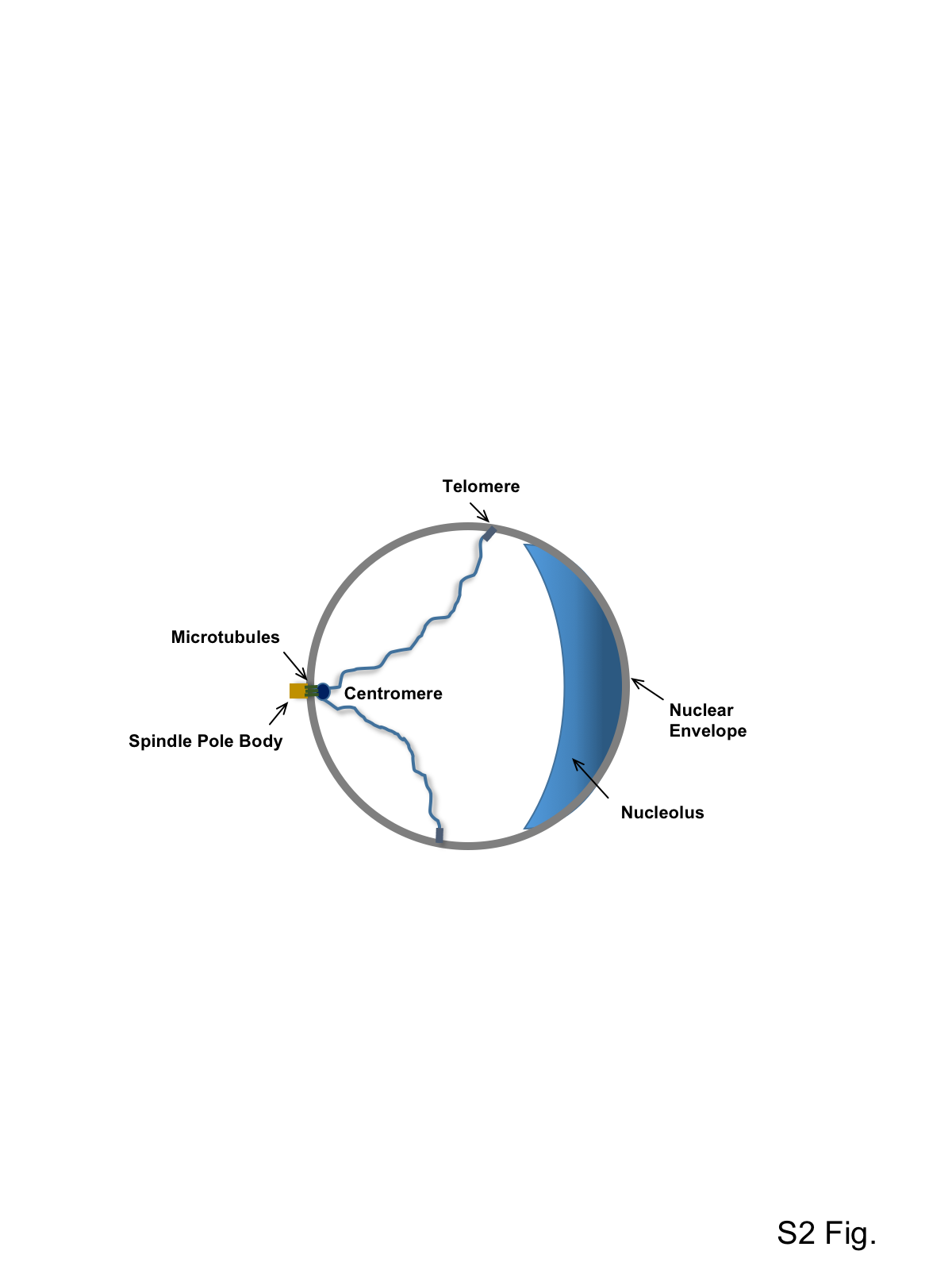

Supplement: S2 Fig — The centromere is tethered to the spindle pole body and the telomeres are clustered at the nuclear envelope. (TIF) [file pone.0180994.s002.tif]

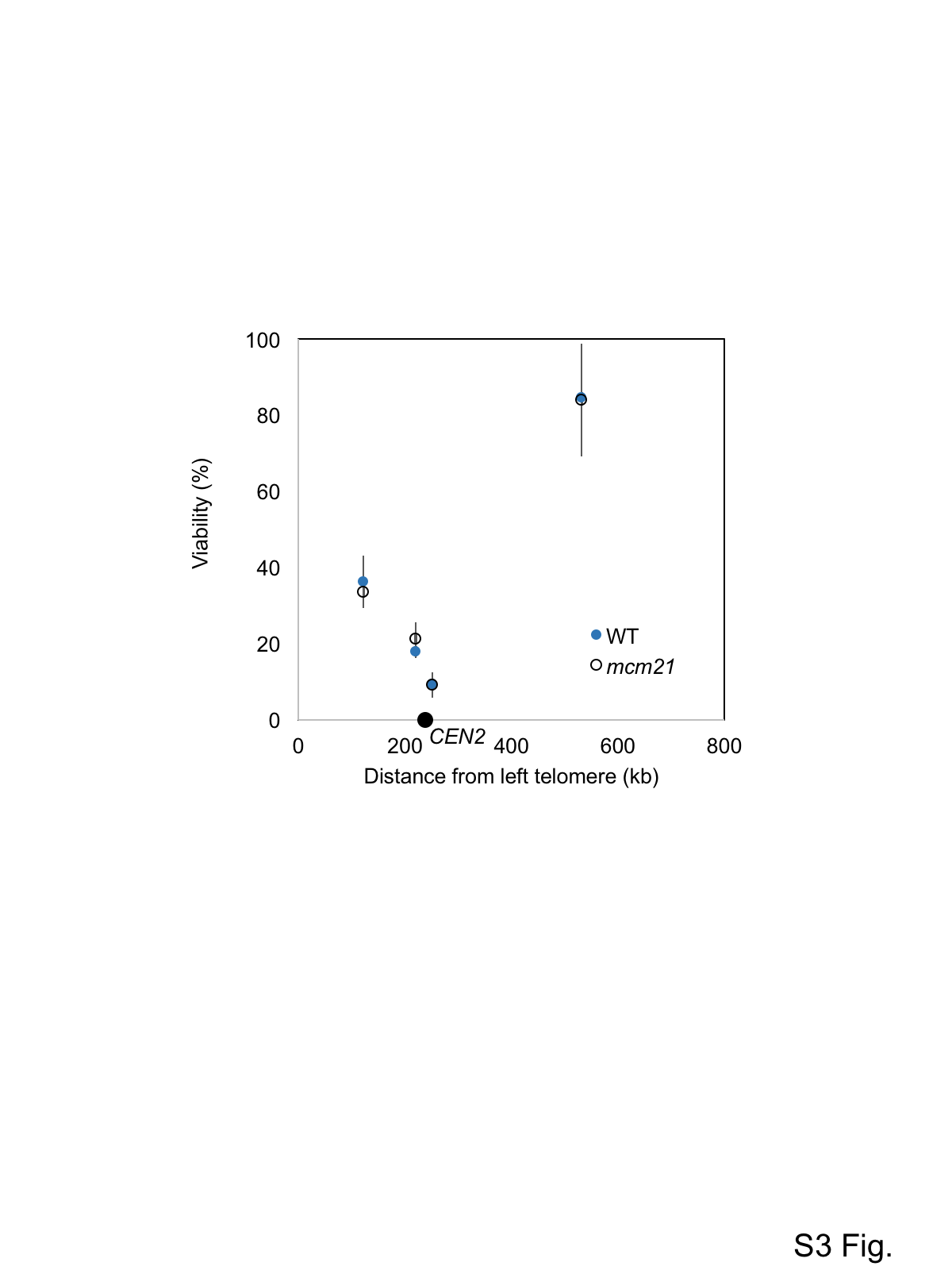

Supplement: S3 Fig — (TIF) [file pone.0180994.s003.tif]

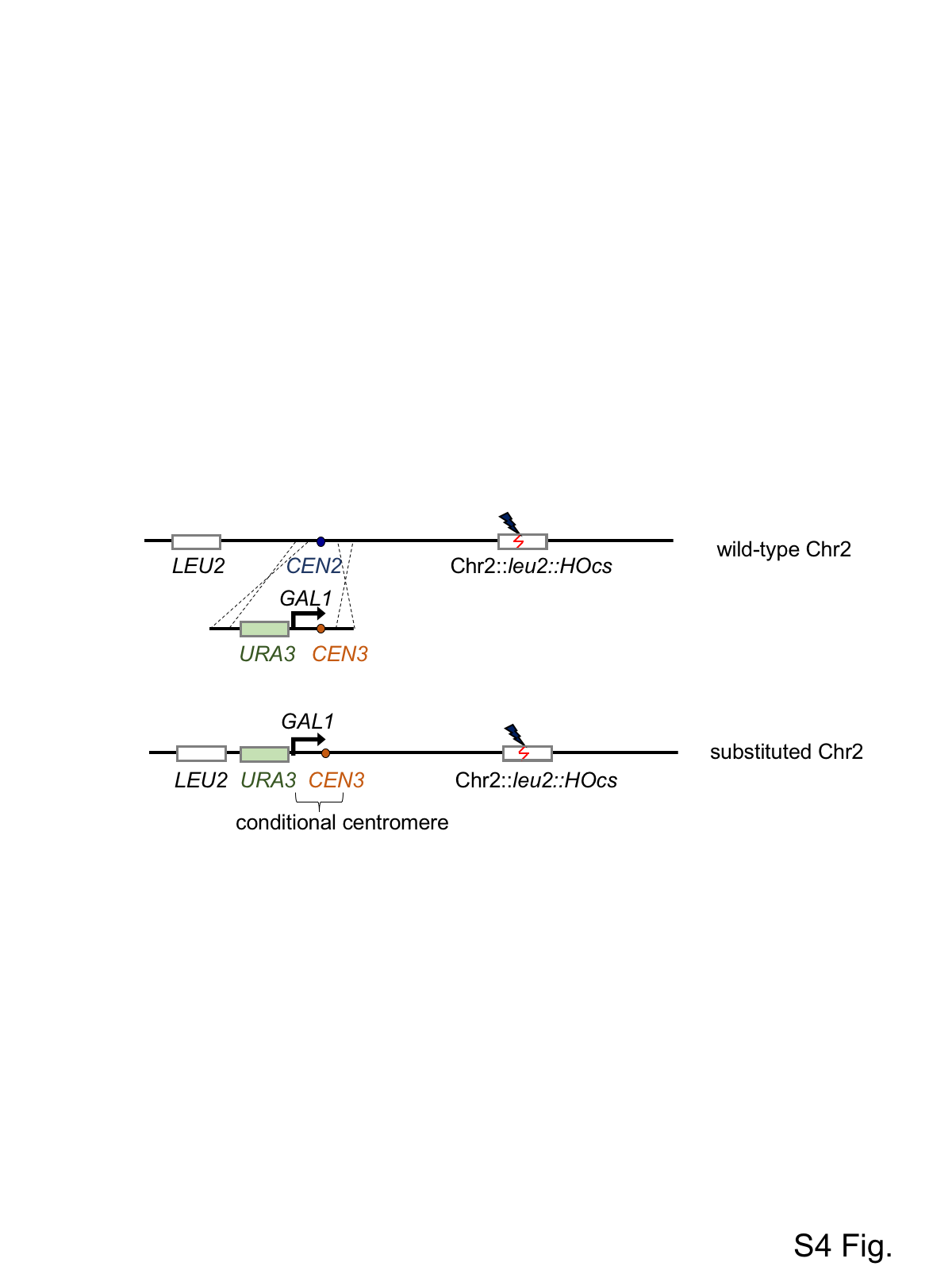

Supplement: S4 Fig — Wild type CEN2 was replaced by a URA3 marked GAL-CEN3 fragment through homologous recombination. The conditional chromosome contains a GAL1 promoter adjacent to CEN3. (TIF) [file pone.0180994.s004.tif]

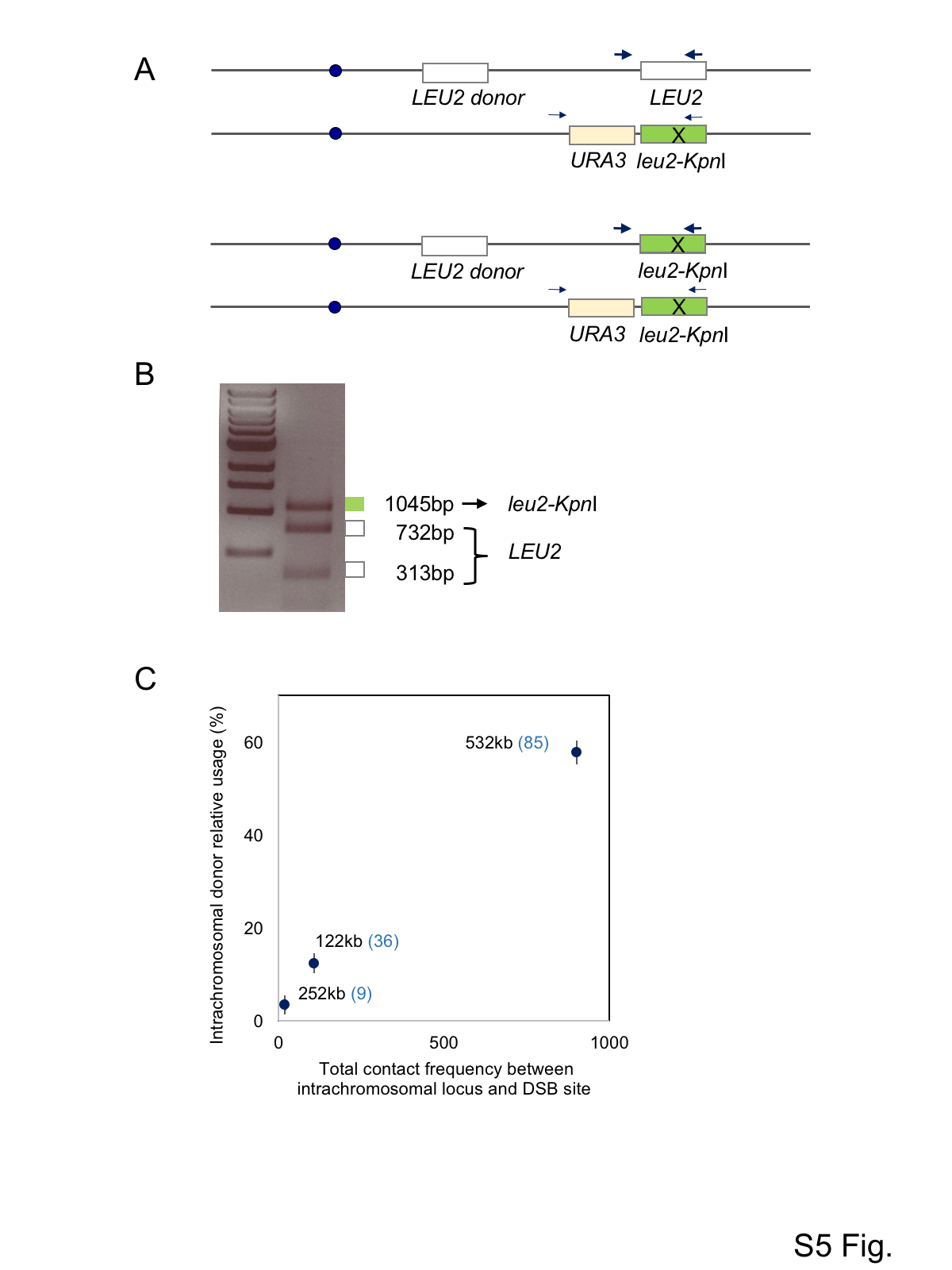

Supplement: S5 Fig — (A) Scheme to assess ectopic and allelic donor usage from a population of cells. The 3kb URA3-leu2-KpnI sequence was excluded in the PCR-based analysis by using short amplification times, as indicated by smaller arrowheads. (B) An example of donor usage measurement on agarose gel (YWW210, 58% intrachromosomal donor usage in diploid strain, 85% viability in haploid strain). The top band (1045 bp) represents leu2-KpnI repair product. The lower two bands (732 bp and 313 bp), digested by KpnI, represent LEU2 repair product. The intrachromosomal donor relative usage (%) was calculated as the intensity of the sum of lower two bands divided by the total intensities of the three bands. (C) Plot of intrachromosomal donor relative usage versus contact frequency (±10 kb around donor and ±25 kb around DSB). The intrachromosomal donor locations and their corresponding viabilities (%) in haploid strains are shown in blue. Error bars indicate one SD from three independent experiments. (TIF) [file pone.0180994.s005.tif]
