# Supplementary material for: Position effects influencing intrachromosomal repair of a double-strand break in budding yeast
Source: PLoS One. 2017 Jul 11;12(7):e0180994. doi: 10.1371/journal.pone.0180994 (PMC5507452; doi:10.1371/journal.pone.0180994)
Supplement: S1 Table — (DOCX) [file pone.0180994.s006.docx]

**S1 Table. List of strains.**

| Strain name | Genotype | Positon of *LEU2* relative to *CEN2* (kb) |
| --- | --- | --- |
| YCSL305 | *ho hml*△::A*DE1 mata*△::*hisG hmr*△::*ADE1 leu2*::KAN *ade3*::GAL::HO *ade1 lys5 ura3-52 trp1* Chr2.625kb::*leu2::HOcs* |  |
| YWW086 | YCSL305, Chr2, 22kb::*LEU2* | -216 |
| YWW090 | YCSL305, Chr2, 122kb::*LEU2* | -116 |
| YWW092 | YCSL305, Chr2, 212kb::*LEU2* | -26 |
| YWW119 | YCSL305, Chr2, 220kb::*LEU2* | -18 |
| YWW113 | YCSL305, Chr2, 252kb::*LEU2* | 14 |
| YWW144 | YCSL305, Chr2, 363kb::*LEU2* | 125 |
| YWW096 | YCSL305, Chr2, 420kb::*LEU2* | 182 |
| YWW099 | YCSL305, Chr2, 532kb::*LEU2* | 294 |
| YWW101 | YCSL305, Chr2, 721kb::*LEU2* | 483 |
| YWW131 | YCSL305, Chr2, 729kb::*LEU2* | 491 |
| YWW147 | YCSL305, Chr2, 742kb::*LEU2* | 504 |
| YWW116 | YCSL305, Chr2, 768kb::*LEU2* | 530 |
| YWW179 | YWW090, *mcm21*::NAT | -116 |
| YWW187 | YWW119, *mcm21*::NAT | -18 |
| YWW185 | YWW113, *mcm21*::NAT | 14 |
| YWW182 | YWW099, *mcm21*::NAT | 294 |
| YWW225 | YWW086, *cen2::URA3-GAL-CEN3* | -216 |
| YWW213 | YWW090, *cen2::URA3-GAL-CEN3* | -116 |
| YWW216 | YWW119, *cen2::URA3-GAL-CEN3* | -18 |
| YWW231 | YWW113, *cen2::URA3-GAL-CEN3* | 14 |
| YWW228 | YWW099, *cen2::URA3-GAL-CEN3* | 294 |
| YWW233 | YWW131, *cen2::URA3-GAL-CEN3* | 491 |
