# Supplementary material for: Position effects influencing intrachromosomal repair of a double-strand break in budding yeast
Source: PLoS One. 2017 Jul 11;12(7):e0180994. doi: 10.1371/journal.pone.0180994 (PMC5507452; doi:10.1371/journal.pone.0180994)
Supplement: S2 Table — (DOCX) [file pone.0180994.s007.docx]

**S2 Table. List of primers.**

| Primer Name | Sequence |
| --- | --- |
| WW006-Ch2, 22kb::NAT p1 | GCACATGTATATATTATACACTACTTATAACTAACCTTCTTCATATATAAgatatcaagcttgcctcgtccccgc |
| WW007-Ch2, 22kb::NAT p2 | ATTTTCTTTCATGATATGATTTAAATGCATTAGTATTATCAGGCATATTGcgacactggatggcggcgttagtatc |
| WW008-Ch2,122kb::NAT p1 | GTTACCCGACTTTCTATATACTACAAATACCGCCAATGATGCAGTGAGCAgatatcaagcttgcctcgtccccgc |
| WW009-Ch2,122kb::NAT p2 | CAGAGATGTTTCTACCTTACTCGATATTGTTTTTGCATTAATTGATATCTcgacactggatggcggcgttagtatc |
| WW010-Ch2,212kb::NAT p1 | GACTACACACCTCCTCTGTCTGGAAGAAATAAATTCTTAGGGTCGACTCCgatatcaagcttgcctcgtccccgc |
| WW011-Ch2,212kb::NAT p2 | CTCAGTCCCAATAATGTTTTATTGTCATATTATACTAAAGTTGAAATGGTcgacactggatggcggcgttagtatc |
| WW033-Ch2,220kb::NAT p1 | CTGTTCAGTAGATATTATATGTAATTATAGCCTCAACAATTTTTCTTGGAgatatcaagcttgcctcgtccccgc |
| WW034-Ch2,220kb::NAT p2 | TCAAACTAAGGAAAATCCAGCCTCTATGGGTGAATAGCTCATCCAATCGAcgacactggatggcggcgttagtatc |
| WW027-Ch2,252kb::NAT p1 | ATCAGGTATTTTGTATTATTTTTTGCGCAACAGCAAGCAAACGCAATAGCgatatcaagcttgcctcgtccccgc |
| WW028-Ch2,252kb::NAT p2 | GTTTGCTGCTATGTATCCTTGCTAAATTTTGATTATATTATGGCATTTATCcgacactggatggcggcgttagtatc |
| WW040-Ch2, 363kb::NAT p1 | AATTCTACGTTCTCAATGGTGGTGTTGAGCTCTAAATTATTTTACAATTTgatatcaagcttgcctcgtccccgc |
| WW041-Ch2, 363kb::NAT p2 | CCTCCTTAAAGGTCATGCTCCTTTTTATGGGTTCTCGTCGTAATAATCCTGcgacactggatggcggcgttagtatc |
| WW014-Ch2,420kb::NAT p1 | CATGTACTGTAAGGCCCAAAAGGGCTCCAGGGATGCCCTTCTACAAAGgatatcaagcttgcctcgtccccgc |
| WW015-Ch2,420kb::NAT p2 | GGGCGGGAGATGTTATTATGTAACGGGGCTTTTATTAGCATGTGAAATGcgacactggatggcggcgttagtatc |
| WW016-Ch2,532kb::NAT p1 | ATAAATTGGAAGGATCTAAGCTAAAGTAAATTATGAATTGAATACTGCCCTgatatcaagcttgcctcgtccccgc |
| WW017-Ch2,532kb::NAT p2 | GAGGATAACAGACTTGTTAAAAAGCATCCTGTCAAAATCTAATTTTTGAcgacactggatggcggcgttagtatc |
| WW018-Ch2,721kb::NAT p1 | ACAAGAATTACTAGCCATATTTTCCCTTGTCAAATTAGAGAAGGAAAAATgatatcaagcttgcctcgtccccgc |
| WW019-Ch2,721kb::NAT p2 | CAGCTCCTATTGATATACTTTTAGTGCCCAAACACGGGATGTTTCAAGTAcgacactggatggcggcgttagtatc |
| WW029-Ch2,729kb::NAT p1 | GTTTTACTAGCCAATATTCATTTTTTTTGAATCGATGTAAAATTTTTGgatatcaagcttgcctcgtccccgc |
| WW030-Ch2,729kb::NAT p2 | GCATGAGCTTTCTGTAGAAATTTAACATTGTGCAATTTTTCTTGTATCcgacactggatggcggcgttagtatc |
| WW042-Ch2, 742kb::NAT p1 | CGAAATAGGTTCAATTCATACTCGAAAAGAATTCGAAGGACGCTCACAAGgatatcaagcttgcctcgtccccgc |
| WW043-Ch2, 742kb::NAT p2 | ATGTGTGTGTTATGAAATGCATACGTTTCATGTTTGCCTAAAATCTCTTTcgacactggatggcggcgttagtatc |
| WW031-Ch2,768kb::NAT p1 | ATAACAAAACATCGAAATCAGAGGATCATTCTAGATCAAAGTGAAATGCCgatatcaagcttgcctcgtccccgc |
| WW032-Ch2,768kb::NAT p2 | AATTATAATAACACTTCACAGCTATTTTCGTATTCCTTTTCATAGTTAAGcgacactggatggcggcgttagtatc |
|  |  |
| WW020-Ch2, 22kb p1 | GTTAATCGCTTCCGTACTCGC |
| WW021-Ch2,122kb p1 | CTGAGAAACGTCACCTAATGTCG |
| WW026-Ch2 212kb p1 | CTACTGCATCAAATCCGTTTGG |
| WW035-Ch2, 220kb p1 | CCCTTATGAACACGCACAGGC |
| WW036-Ch2, 252kb p1 | CGGTTACTTTGTGACCCTTTGC |
| WW044-Ch2, 363kb p1 | CTAGTCACAAACATCCGCATAGGC |
| WW023-Ch2, 420kb p1 | GAAAGATTCTAGGCCGGGTCC |
| WW024-Ch2, 532kb p2 | GATGAATGTGCACATGCTGTCG |
| WW025-Ch2, 721kb p1 | CTTTAAGGAACTTTACTCCGCG |
| WW037-Ch2, 729kb p1 | CAACGGACCGGGTAAAAAGCG |
| WW045-Ch2,742kb p1 | CGATATTGGGTCATTCTCGCCTG |
| WW038-Ch2, 768kb p1 | AGAAAATCATCATCAAATAGCCGCC |
| NATp1B | AGGCGCTCTACATGAGCA |
| Leu2p18B | CCAAATAGGCAATGGTGGCT |
|  |  |
| WW046-Mcm21::MX p1 | GGATAATGAGGGGAAAATGTGATTTCTTTACTTGAGCAATTCTTTCAAACCCAGATCTGTTTAGCTTGCC |
| WW047-Mcm21::MX p2 | CAGAGAAAATTAGCTCTATCCTCTTTCTATAAAGTATATTTTTGTTAACATtggatggcggcgttagtatc |
| WW048-Mcm21 DS p1 | GGAAATGGGATGGTGGGTTTGAC |
| WW049-Mcm21 ORF p1 | CCAATGGGGGACTAGTGATATCTC |
| WW057-cen2::GAL-CEN3 p4 | CGGTGAACAATTTTTTGCAAGAAATATATTGATACTTCTTGTTGAAATATTGATGTCGGCGATATAGGCG |
| WW058-cen2::GAL-CEN3 p5 | ACTAATATCAATAATAATAAATTAATCTTGAGCAAATTGATCCTACATAAGAGGCCCTTTCGTCTTCAAG |
| WW052-Chr2,237kb p1 | CCATCTTCATTTCAAATAAGAGAGC |
| WW053-Chr2,238kb p1 | GGGCATTTCAGTAACATCTTCAAAC |
| Leu2p18B | CCAAATAGGCAATGGTGGCT |
| Mcm7p3 | TGCGAAGCACAGGTGTTCACTTTC |
